# Effect of Promising Sustainable Nano-Reinforcements on Polysulfone/Polyvinylpyrrolidone-Based Membranes: Enhancing Mechanical Properties and Water Filtration Performance

**DOI:** 10.3390/polym16243531

**Published:** 2024-12-18

**Authors:** Seren Acarer Arat, İnci Pir, Mertol Tüfekci, Nurtaç Öz, Neşe Tüfekci

**Affiliations:** 1 Department of Environmental Engineering, Istanbul University-Cerrahpaşa, Avcilar, Istanbul 34320, Turkey; nese@iuc.edu.tr; 2 Faculty of Mechanical Engineering, Istanbul Technical University, Gumussuyu, Istanbul 34437, Turkey; pirin@itu.edu.tr; 3 Centre for Engineering Research, University of Hertfordshire, Hatfield AL10 9AB, UK; m.tufekci@herts.ac.uk; 4 School of Physics, Engineering and Computer Science, University of Hertfordshire, Hatfield AL10 9AB, UK; 5 Environmental Engineering Department, Engineering Faculty, Sakarya University, Esentepe Campus, Sakarya 54050, Turkey

**Keywords:** membrane, polysulfone, polyvinylpyrrolidone, cellulose nanocrystal, cellulose nanofibre, characterisation, water flux, antifouling ability, organic matter, modelling

## Abstract

In this study, polysulfone/polyvinylpyrrolidone (PSf/PVP, 20 wt%/5 wt%)-based ultrafiltration (UF) membranes reinforced with different ratios (0.5 and 1 wt%) of cellulose nanocrystals (CNCs) and cellulose nanofibres (CNFs) were prepared by the phase inversion method. The effect of CNC, CNF, and CNC-CNF reinforcement on the morphology, roughness, crystallinity, porosity, average pore size, mechanical properties, and filtration performance of PSf/PVP-based membrane was investigated. Distilled water and surface water (lake water) fluxes of the membranes were determined at 3 bar using a dead-end filtration system. The distilled water flux of the fouled–hydraulic cleaned membranes was determined, and scanning electron microscopy (SEM) images of the fouled–cleaned membranes were examined. The flux recovery ratio (FRR) and fouling parameters were calculated to examine the fouling behaviour of the membranes. The mechanical properties of the membranes were modelled by the Mori–Tanaka, finite element, Voigt–Reuss, self-consistent scheme, and Halpin–Tsai methods using Digimat and/or analytically. In addition, the von Mises equivalent stress distributions of the nanocomposites were presented. Among the investigated membranes, PSf/PVP/CNC-0.5 had the highest distilled water flux (475.5 ± 17.77 L/m^2^.h), PSf/PVP/CNF-1 exhibited the stiffest behaviour with an elasticity modulus of 70.63 ± 3.15 MPa, and PSf/PVP/CNC-1 had the best organic matter removal efficiency. The finite element was the most successful modelling method for estimating the mechanical properties of nanocellulose-reinforced flat sheet membranes.

## 1. Introduction

Membrane filtration is a modern treatment option widely used in potable water production and wastewater treatment due to its superior properties [1]. A reliable and rapid supply of potable water and high-quality water for agricultural irrigation and industrial processes has become increasingly important due to the ever-increasing demand for fresh water [2]. Membrane processes offer a solution for drinking water treatment as well as for reducing the environmental impact of large volumes of industrial wastewater generated worldwide due to rapid industrial growth [3]. Membrane processes are good alternatives for increasing the reusability of process water in industries or treating industrial wastewater with high efficiencies [4]. Moreover, the application of membrane processes in the treatment of landfill leachate, which is considered a strong wastewater, minimises the risk of pollutant release from leachate into the environment [5]. Membrane technologies provide superior performance in minimising the negative environmental impact of wastewater by removing parameters such as solids, organic matter, microorganisms, turbidity, heavy metals, microplastics, oil and grease, nitrogen, phosphorus, and salts from surface water, wastewater, and landfill leachate with high efficiency [6,7,8,9,10].

The advantages of membrane processes for water filtration applications include high-quality water production, requiring fewer chemical substances and energy consumption, low maintenance costs, and easy scale-up [11]. Membranes are mainly categorised into two groups according to their materials: polymeric membranes and inorganic membranes. Polymeric membranes are widely used in water filtration due to their easy production, easy processability, and low cost [12]. Polysulfone (PSf) is widely used in the production of membranes due to its membrane-forming ability, ability to dissolve in organic solvents commonly used in membrane production, and superior thermal, chemical, and mechanical stability [13,14]. PSf membranes are frequently preferred in microfiltration (MF), ultrafiltration (UF), and membrane bioreactor (MBR) applications [15,16,17]. PSf membranes are also used as sub-layers in membranes [18]. However, the hydrophobic nature of PSf makes PSf-based membranes prone to fouling, reducing the lifespan and performance of the membrane [18,19]. Various nanomaterials have been blended into the PSf-based membrane matrix in many studies to enhance the properties and improve the filtration performance of the PSf membrane [20,21,22,23,24].

Analysing the mechanical properties of membranes produced for application in water treatment plays an important role not only in membrane design but also in predicting membrane failures during filtration [25]. Investigating the mechanical properties of membranes also contributes to determining the maximum pressure that can be applied to the membrane so that it can be operated without deformation, fracture, or cracking during filtration. Since both mechanical fatigue and the chemicals used for cleaning the membranes accelerate the mechanical deformation of the membrane, membranes with good mechanical properties can be used longer with less risk of deformation [25]. The improved mechanical properties of the membrane result in a reduction in the potential for deformation under pressure [26], thereby contributing to the uninterrupted water flux. Additionally, although large pores allow water to be easily filtered through the membrane, large pores also facilitate the transfer of contaminants from the feed to the membrane permeate [27]. Since membranes whose pores expand during filtration due to low mechanical properties cannot provide a stable water flux and separation performance [28], mechanical properties must be taken into account in the production of membranes. Therefore, high water flux to reduce energy demand as well as high mechanical strength to ensure a long lifetime and stable filtration are essential for membranes used in water treatment [29,30].

Cellulose is a natural, abundant, sustainable, eco-friendly biopolymer [31]. Nanocellulose is a nanomaterial that is derived from cellulose and has high hydrophilicity, biocompatibility, and superior mechanical properties, particularly better mechanical strength [31,32]. Nanocellulose is primarily divided into three classes: cellulose nanocrystal (CNC), cellulose nanofibre (CNF), and bacterial nanocellulose (BC) [33]. CNCs and CNFs are plant-derived, hydrophilic nanomaterials containing abundant hydroxyl groups (-OH) [34,35,36]. CNCs are a type of nanocellulose obtained through acid hydrolysis [37]. CNFs are nanocellulose derivatives produced through mechanical and chemical processes [38]. Nanocellulose obtained from plants is a promising option for enhancing the properties of polymer-based nanocomposite materials [39].

Recent studies have shown that the incorporation of CNC [40,41] and CNF [42,43,44,45] into polymer-based membranes improves the structural properties and filtration performance of the membrane. Fahmy et al. obtained CNC by hydrolysing bleached pulp with sulfuric acid and incorporating CNC into a membrane based on 20 wt% PSf. In the study, the incorporation of CNC into the PSf membrane increased the pure water flux, oil–water separation efficiency, and oil emulsion flux of the membrane [46]. Rasid et al. (2022) characterised asymmetric nanocomposite membranes produced by incorporating 1 wt% CNC into a 15 wt% PSf-based membrane, and they reported that CNC incorporation resulted in changes in pores in the internal structure of the membrane, surface wettability, porosity, flux performance, and copper removal efficiency [47]. Alasfar et al. investigated the effect of 0.1–0.5% CNF incorporation on the properties and performance of an 18 wt% PSf-based membrane. Up to 0.3 wt% CNF incorporation, the porosity and contact angle of the neat PSf membrane decreased, while the average pore size, flux, elasticity modulus, and elongation at break increased [45]. In a study by Ding et al., the effect of 0.2–1.2% CNF incorporation into PSf/polyvinyl pyrrolidone (PVP) membrane based on 18 wt% PSf and 1% PVP was investigated. The results of the study showed that the elasticity modulus of the PSf/PVP membrane doped with 1 wt% CNF (217 ± 8.31 MPa) was higher than that of the PSf/PVP membrane (157 ± 13.16 MPa). In addition, the pure water flux of the PSf/PVP/CNF membrane (~680 L/m^2^.h) was significantly higher than that of the PSf/PVP membrane (319 L/m^2^.h) [48].

The amount and properties of the polymer (molecular weight, hydrophilic/hydrophobic properties, etc.), the amount and type of solvent, polymer–solvent interaction, and the type and amount of other additives such as nanomaterials in the membrane casting solution significantly affect the properties of the casting solution, and kinetic and thermodynamic processes during phase inversion [49,50,51]. Furthermore, membrane casting conditions and coagulation bath conditions affect the properties of the final membrane [52]. Since all these factors lead to different membrane properties and performance, the properties and performance of polymer-based membranes produced from casting solutions of different compositions need to be reported in the literature to overcome the problems encountered in membrane processes and to further improve the performance of membranes.

In this study, CNC (0.5 and 1 wt%)-, CNF (0. 5 wt%)-, and CNC-CNF (0.5 wt%)-reinforced polymer-based membranes were fabricated by non-solvent (water)-induced phase inversion method using PSf with a molecular weight of ~35,000 Da as the polymer, dimethyl formamide (DMF) as the solvent, and 5 wt% PVP as the pore-forming agent, and the characterisation and performance results of the produced membranes were reported. To the best of the authors’ knowledge, there is no study in the literature investigating the effect of different rates of CNC and CNF reinforcement on the properties and filtration performance of polymer-based membranes consisting of 20 wt% PSf and 5 wt% PVP. In this study, the effects on the morphology, crystallinity, mechanical properties, flux performance, fouling resistance, and ultraviolet absorbance at 254 nm (UV254) and total organic carbon (TOC) removal efficiency of PSf/PVP-based membrane were experimentally investigated. In addition, the success of different modelling techniques in estimating the mechanical properties of nanocellulose (CNC, CNF, and CNC-CNF)-reinforced nanocomposite membrane was investigated using the mechanical test results of PSf/PVP-based membrane and the properties of nanomaterials. This study fills the research gap by experimentally evaluating the effects of CNC and CNF at different ratios on PSf/PVP membranes and integrating mechanical tests with modelling techniques to predict membrane performance. This dual focus on experimental and predictive modelling is unique for polymer-based nanocomposite membranes and provides a comprehensive understanding of their potential applications. Furthermore, to the best of the authors’ knowledge, this is also the first study to evaluate the UV254 absorbance and TOC removal performance of CNC- and CNF-reinforced PSf/PVP membranes and highlight their potential for advanced water treatment applications.

## 2. Materials and Methods

### 2.1. Materials

PSf with an average molecular weight of ~35,000 Da was obtained from Aldrich. DMF used as solvent was purchased from Carlo Erba. PVP in powder form with an average molecular weight of 40,000 Da was purchased from Sigma-Aldrich. The width of CNC and CNF used in nanocomposite membrane production was 10–20 nm. The lengths of CNC and CNF were 300–900 nm and 2–3 μm, respectively. All materials were utilised as received, without any additional purification, during the membrane fabrication process.

### 2.2. Production of PSf/PVP-Based Flat Sheet Membranes

**Figure 1:**
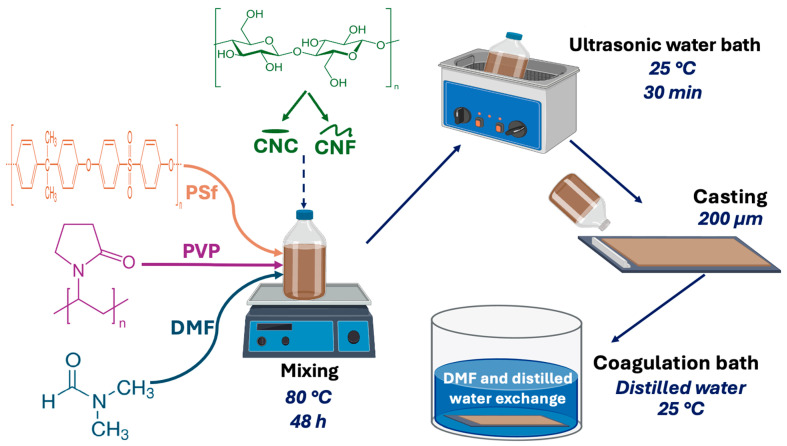
The main production steps of PSf/PVP-based membranes.

In this study, flat sheet membranes of PSf/PVP-based membranes were produced by the previously reported phase inversion method [53]. The PSf, PVP, CNC, and CNF used in membrane production were kept in an oven (Nuve EN 500) set at 45 °C for 2 h to remove possible existing moisture. To prepare the PSf/PVP membrane casting solution, 75 wt% DMF was added into dry glass bottles, and then DMF was stirred in a heated magnetic stirrer (Wisd, MSH20A) at 80 °C for 5 min. In the next step, 5 wt% PVP was added to DMF, and stirring was continued until the PVP was completely dissolved. Then, 20 wt% PSf was added to the solution, and the solution was stirred at 80 °C for 48 h. After obtaining a homogeneous solution, the bottles were placed in an ultrasonic water bath (Weightlab Instruments) at 25 °C for 30 min in degassing mode to remove air bubbles from the solution. In the next step, the solution was poured onto a dry and plain glass plate. Using a 200 μm thick casting knife (TQC Sheen, VF2170-261), the solution was spread on the glass plate to obtain a polymeric film. It is worth noting that in the non-solvent-induced phase inversion method, the casting thickness affects the rate of liquid–liquid exchange in the coagulation bath, leading to differences in the properties of the final membrane [54]. A 200 μm casting thickness is widely used in the production of flat sheet membranes using the non-solvent-induced phase inversion method [53,55]. To make the properties of the membranes produced in this study more comparable with the properties of the membranes in the literature studies, a casting thickness compatible with the literature was preferred in the production of flat sheet membranes. Afterward, the glass plate was immersed in a coagulation bath containing only distilled water at 25 °C. As a result of the liquid–liquid exchange between the solvent (DMF) in the polymeric film and the non-solvent (distilled water) in the coagulation bath, a flat sheet membrane was obtained in solid form. The front and back surfaces of the membrane were thoroughly washed with distilled water to remove impurities from the membrane surfaces produced by the phase inversion method. The production of CNC-, CNF-, and CNC-CNF-reinforced PSf/PVP membranes followed the same mixing, casting, and coagulation conditions as those used for PSf/PVP membranes. All produced membranes were stored in distilled water in separate containers. Figure 1 shows the main production steps of PSf/PVP-based flat sheet membranes. Table 1 shows the composition of the casting solutions of PSf/PVP and nanocomposite PSf/PVP membranes.

### 2.3. Membrane Characterisation

The viscosity of the membrane casting solutions was measured by a viscometer at 22 °C.

The surface morphology of the membranes was analysed using a scanning electron microscope (SEM, Philips XL 30S FEG, Tokyo, Japan). Prior to SEM analysis, the membranes were dried at room temperature for 48 h. The surface of the dry membranes was rendered conductive through the application of a gold coating, applied at a current of 10 mA for a period of 90 s using a coating device (Quorum SC7620 (Woonsocket, RI, USA). The surface morphology of the produced membranes was examined at 2000× magnification at 5 kV, while the surface morphology of the fouled–cleaned membranes after filtration was examined at 5000× magnification at 5 kV.

Atomic Force Microscopy (AFM, Digital Instruments Nanoscope IV, Bedford, TX, USA) was used to examine the surface roughness of the membranes. AFM analysis was conducted in contact mode utilising a Bruker silicon nitride probe. Three roughness parameters, mean roughness (Ra), root mean square roughness (Rrms), and mean difference between the highest peaks and lowest valleys (Rz), were determined by AFM analysis.

The X-Ray Diffraction (XRD) method was used to determine the crystalline properties of the membranes. XRD patterns of the membranes were determined using an X-ray diffractometer (Bruker D8 Advance, Ettlingen, Germany) equipped with Cu Kα (k = 1.54 Å) radiation (40 kV, 40 mA). XRD patterns were obtained over a diffraction angle range of 2θ = 3–70°.

The porosity (*P*) of the membranes was determined by the gravimetric method, and Equation (1) was used to calculate the membrane porosities [53].

(1) $$P\;\left(\%\right)=\frac{m_{w}-m_{d}}{A\;t\;\rho \;}\times 100$$

where *m_w_* and *m_d_* are the wet and dry weights of the membrane (g), *A* is the membrane area (cm^2^), *t* is the membrane thickness (cm), *ρ* is the density of water (0.998 g/cm^3^).

After the porosity of the membranes was calculated, the average pore size (r) of the membranes was calculated using Equation (2) (Guerout–Elford–Ferry equation) [53].

(2) $$r=\sqrt{\frac{\left(2.9-1.75P\right)\times 8\eta lQ}{P\times A\times \Delta P}}$$

where *η* is the viscosity of water (8.9 × 10^−4^ Pa.s), *l* is the membrane thickness (m), *Q* is the permeate volume per unit time (m^3^/s), *A* is the effective membrane area (m^2^), and Δ*P* is the operating pressure (0.3 MPa).

**Figure 2:**
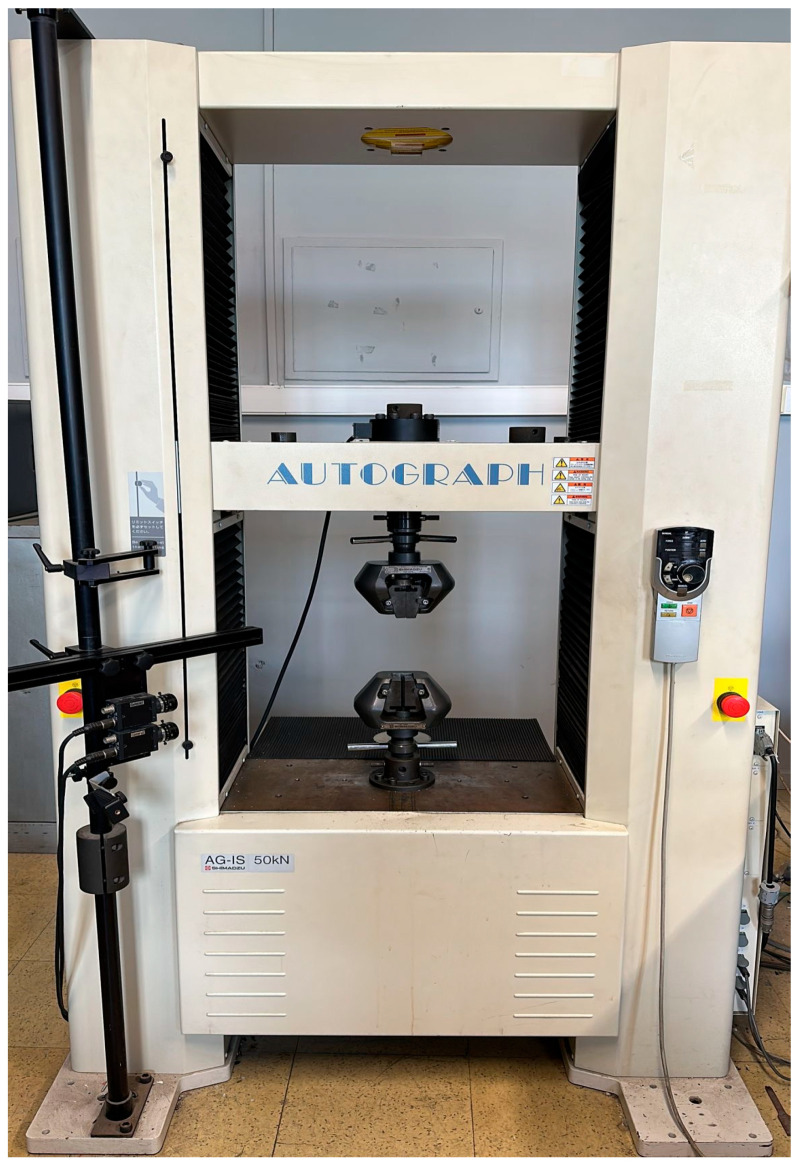
Test machine for tensile testing of membranes.

Stress–strain curve and mechanical properties of membranes (elasticity modulus, tensile strength, and elongation at break) were determined by tensile test. Tensile tests were conducted using a universal testing machine (Shimadzu AG-IS 50 kN) (Figure 2). Tensile tests were conducted under a strain rate of 1% per minute.

### 2.4. Water Flux and Rejection Performance of Membranes

The water flux performance of the membranes was performed using a dead-end filtration system (Tin Mühendislik). Samples with a diameter of 5 cm were cut from the membranes using scissors. The cut circular membrane samples were placed in the dead-end filtration system. Following the placement of the magnetic apparatus within the filtration cell, 300 mL of distilled water was added to the filtration cell. Nitrogen gas (N_2_ gas) was used to filter the water through the membrane. Water was filtered through the membranes at 3 bar using N_2_ gas. Membrane permeates were collected in clean beakers of 250 mL volume on an AND EJ-610 precision balance. Using WinCT-RSWeight software (version 6.02), data were transferred to a computer every minute, and a time–weight plot was generated for 15 min.

To determine the flux performance of the membranes for surface water, a water sample was collected from Terkos Lake in Istanbul, Turkey. In the filtration process, the same conditions applied for distilled water were applied to lake water. The distilled water and lake water filtered membrane samples were physically cleaned by immersing them in distilled water for 15 min. The flux performance of the physically cleaned (fouled–cleaned) membranes was determined for distilled water at 3 bar. The flux performance of clean membranes for distilled water, flux performance of clean membranes for lake water, and flux performance of fouled–cleaned membranes for distilled water was measured using a dead-end filtration technique at 3 bar and room temperature. Equation (3) was used to determine the flux performance of the membranes [53].

(3) $$J=\frac{V}{A\times \Delta t}$$

where *J* is water flux (L/m^2^.h). *A* is membrane area (m^2^). ∆*t* is time (h). *V* is permeate volume (L).

To determine the UV254 removal efficiency of the membranes from lake water, feed (lake water) and membrane permeates were analysed at 254 nm wavelength using a UV254 spectrophotometer (Hach-Lange DR5000, Düsseldorf, Germany). TOC analysis in the feed and membrane permeate was performed using a TOC analyser (Sievers 5310C, Auburn, IL, USA).

### 2.5. Antifouling Performance of Membranes

To investigate the antifouling performance of the membranes, the total fouling ratio (Rt), reversible fouling ratio (Rr), irreversible fouling ratio (Rir), and flux recovery ratio (FRR) of the membranes were calculated using Equations (4)–(7) [53].

(4) $$\mathrm{Rt}\;\left(\%\right)=\frac{j_{w1}-j_{l}}{j_{w1}}\times 100$$

(5) $$Rr\;\left(\%\right)=\frac{j_{w2}-j_{l}}{j_{w1}}\times 100$$

(6) $$Rir\;\left(\%\right)=\frac{j_{w1}-j_{w2}}{j_{w1}}\times 100$$

(7) $$FRR\;\left(\%\right)=\frac{j_{w2}}{j_{w1}}\times 100$$

where *j_w_*_1_ is the distilled water flux of the clean membrane. *J_w_*_2_ is the distilled water flux of the fouled–cleaned membrane. *J_l_* is the lake water (surface water) flux of the clean membrane.

### 2.6. Estimation of Mechanical Properties of Nanocomposite Membranes with Models

The mechanical properties of PSf/PVP-based nanocomposite membranes were estimated by different modelling methods using the data obtained from the mechanical tests of PSf/PVP membrane and the properties of nanocellulose reinforcements (CNC and CNF). The model results were compared with the experimental tensile test results of nanocomposite membranes, and the success of the models in estimating the mechanical properties of nanocomposite membranes was investigated. In this study, the mechanical properties of membranes were estimated using the Mori–Tanaka mean-field homogenisation method, finite element method, self-consistent scheme method, Voigt–Reuss method, and Halpin–Tsai method. Among these five modelling methods, Mori–Tanaka mean-field homogenisation and finite element modelling studies were carried out using Digimat (version 2023.2), a piece of software that is used for the mechanical modelling of composite materials. On the other hand, the self-consistent scheme, Voigt–Reuss, and Halpin–Tsai models were computationally based.

In the Mori–Tanaka mean-field homogenisation method, the elasticity modulus obtained from the experimental tensile test of the PSf/PVP membrane was entered into the Digimat MF tool for the matrix (PSf/PVP) properties. Poisson’s ratio, density, and elasticity modulus values for nanocellulose reinforcements were entered as 0.25, 1.5 g/cm^3^, and 80 GPa, respectively [53]. The aspect ratios for CNF and CNF were introduced into the Digimat MF toolbox as 85–130 and 60–100, respectively. In the Mori–Tanaka mean-field homogenisation method, both the membrane matrix and nanocellulose reinforcements were assumed to have a homogeneous and isotropic structure as well as linear elastic behaviour. CNC, CNF, and CNC-CNF reinforcements were randomly distributed in the membrane matrix.

In the finite element model, nanocellulose-reinforced, isotropic cubic representative volume elements (RVEs) were generated in Digimat FE. The RVEs were divided into tetrahedral elements. In the finite element method, the values for the matrix and nanocellulose reinforcements were the same as those used in the Mori–Tanaka mean-field homogenisation method.

Stress along the composite material (membrane) in the Voigt model and strain along the composite material in the Reuss model were assumed to be constant. An upper bound (*E_Voigt_*) and lower bound (E*_Reuss_*) were calculated for the modulus of elasticity of the nanocomposite membrane in the Voigt model and Reuss model, respectively [56]. *E_Voigt_* and *E_Reuss_* values were calculated using Equations (8) and (9), respectively.

(8) $$E_{\mathrm{Voigt}}=E_{f}V_{f}+E_{m}\left(1-V_{f}\right)$$

(9) $$E_{\mathrm{Reuss}}=\frac{1}{\frac{V_{f}}{E_{f}}+\frac{1-V_{f}}{E_{m}}}$$

where *E*_*f*_ is the elasticity modulus of the nanomaterials. *E*_*m*_ is elasticity modulus of membrane matrix. *V*_*f*_ is the volume fraction of the nanomaterials in the membrane matrix.

The self-consistent scheme method aims to simulate the micro-level properties of the composite material and their effects on the macro-scale elastic behaviour. In the self-consistent scheme method, the reinforcement phase (CNC, CNF, CNC-CNF) and the matrix (PSf/PVP) phase of the membrane are considered phases that interact in a general structure but are considered homogeneous in themselves. In this method, the effective modulus of elasticity (*E_eff_*) was calculated using Equation (10) [57,58].

(10) $$E_{\mathrm{eff}}=E_{m}\left(1+\frac{3V_{f}\left(E_{f}/E_{\mathrm{eff}}-1\right)}{1+V_{f}\left(E_{f}/E_{\mathrm{eff}}-1\right)}\right)$$

where *E_f_* and *E_m_* are the elasticity modulus of the fibres and matrix. *V_f_* is the volume fraction of the fibres in the membrane.

The Halpin–Tsai model was developed to estimate the mechanical behaviour of composites based on the properties and geometry of their components. Factors such as matrix and reinforcement properties, matrix-reinforcement interfacial interactions, and reinforcement orientation play an important role in the Halpin–Tsai model. For the Halpin–Tsai model, the effective longitudinal (*E*_*eff*,*long*_) and effective transverse (*E*_*eff*,*trans*_) elasticity modulus were calculated using Equations (11) and (12) [58,59].

(11) $$E_{\mathrm{eff},\mathrm{long}}=E_{m}\left(1+\frac{\eta_{L}V_{f}\left(E_{f}/E_{m}-1\right)}{1-\eta_{L}V_{f}\left(E_{f}/E_{m}-1\right)}\right)$$

where *E_m_* is elasticity modulus of the matrix. *η_L_* is a parameter related to the aspect ratio of the nanomaterials.

(12) $$E_{\mathrm{eff},\mathrm{trans}}=E_{m}\left(1+\frac{\eta_{T}V_{f}\left(E_{f}/E_{m}-1\right)}{1-\eta_{T}V_{f}\left(E_{f}/E_{m}-1\right)}\right)$$

where the parameter *η_T_* is associated with the transverse aspect ratio of the nanomaterials.

## 3. Results and Discussion

### 3.1. Viscosity of Membrane Casting Solutions

**Figure 3:**
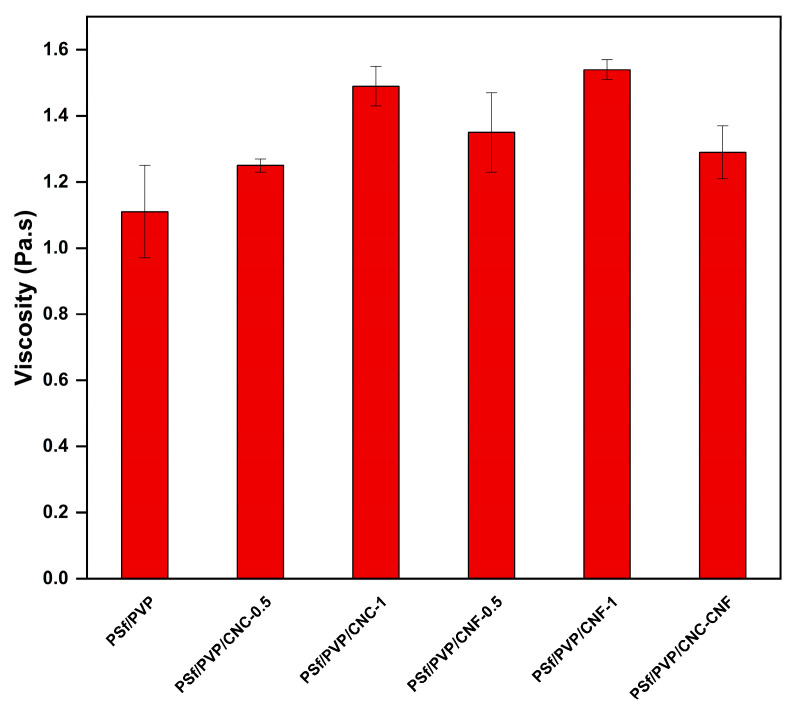
The viscosity of casting solutions prepared for the production of PSf/PVP-based membranes.

In the production of flat sheet membranes via the non-solvent-induced phase inversion method, the viscosity of the membrane casting solution significantly affects the exchange rate between solvent and non-solvent [60]. The viscosity of the membrane casting solution varies depending on the amount of polymer, properties of the polymer, type of solvent, amount of PVP, properties of PVP, and temperature of the casting solution [49,61,62,63,64,65]. Figure 3 shows the viscosity values of the casting solutions at 22 °C prepared to produce PSf/PVP-based membranes. The casting solution of the PSf/PVP membrane had the lowest viscosity value of 1.11 ± 0.14 Pa.s. With the inclusion of CNC and CNF in the PSf/PVP/DMF solution, the viscosity of the solution increased, and the viscosity of the casting solutions prepared for nanocomposite membrane production varied between 1.25 ± 0.02 and 1.54 ± 0.03 Pa.s. When the amount of CNC and CNF in the membrane casting solution increased from 0.5 to 1 wt%, the viscosity of the membrane increased further due to the casting solutions containing more solids. CNF-doped casting solutions had a higher viscosity than CNC-doped casting solutions at the same concentration. Similarly, it has been reported that CNC has a lower viscosity than CNF in suspensions at the same concentration [66].

### 3.2. Membrane Surface Morphology

**Figure 4:**
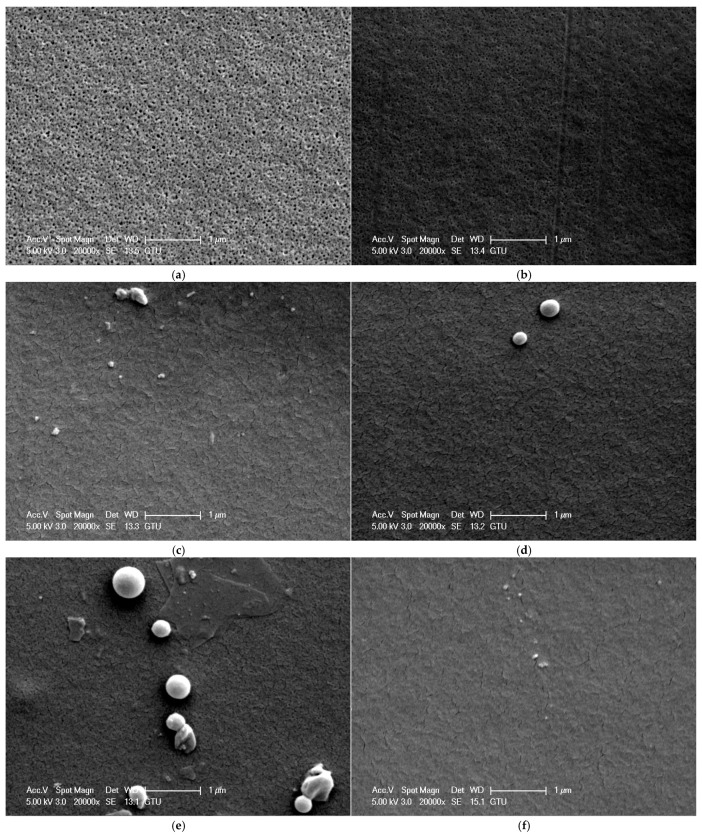
SEM surface images of PSf/PVP-based membranes: (**a**) PSf/PVP, (**b**) PSf/PVP/CNC-0.5, (**c**) PSf/PVP/CNC-1, (**d**) PSf/PVP/CNF-0.5, (**e**) PSf/PVP/CNF-1, and (**f**) PSf/PVP/CNC-CNF.

The surface morphology of membranes affects the membrane’s water flux, separation, and antifouling performances. Figure 4 shows the SEM surface images of PSf/PVP-based flat sheet membranes produced. The surface porosity and the size of the pores on the surface of the PSf/PVP membrane (Figure 4a) were higher than those of the nanocellulose-reinforced membranes (Figure 4a–f). This can be explained by the low viscosity of the PSf/PVP membrane casting solution. Lower membrane casting solution viscosity leads to faster liquid–liquid exchange during phase inversion, while higher viscosity leads to slower liquid–liquid exchange [67]. Faster liquid–liquid exchange leads to a final membrane morphology with higher porosity and/or pore size, while slower liquid–liquid exchange leads to a final membrane morphology with a dense surface [68]. The higher viscosity of the casting solutions containing nanocellulose compared to the casting solution of PSf/PVP membrane led to the formation of membranes with denser structures. The large pores on the upper surface of the PSf/PVP membrane disappeared (Figure 4a), especially with the addition of high amounts of CNC into the membrane (Figure 4b,c). As for CNF reinforcement, the surface porosity of the PSf/PVP membrane decreased significantly with low and high CNF reinforcement in the PSf/PVP membrane (Figure 4d,e). CNF agglomerates were present on the surface of the PSf/PVP/CNF-1 membrane, which was related to the poor dispersion of high amounts of CNF in the membrane casting solution (Figure 4e). Some layers were also observed on the surface of the PSf/PVP/CNF-1 membrane, indicating that high CNF reinforcement prevented the smooth surface formation of flat sheet membranes produced via the phase inversion method (Figure 4e). Combining CNC and CNF reinforcement significantly reduced the porosity of the PSf/PVP membrane (Figure 4f). However, the presence of very small agglomerates on the surface was also observed (Figure 4f). When the amount of CNC and CNF reinforcement in the membrane increased, the surfaces of the membranes had a denser structure with less porosity due to the increased casting solution viscosity. There was no agglomeration on the surfaces of the PSf/PVP and PSf/PVP/CNC-0.5 membranes. The absence of agglomeration on the surface of both membranes was related to the fact that the PSf/PVP membrane did not contain any nanocellulose reinforcement and the low amount of CNC reinforcement was well dispersed in the PSf/PVP/CNC-0.5 membrane. However, agglomeration occurred on the surface of the membranes at 1 wt% CNC, 0.5 wt% CNF, 1 wt% CNF, and 0.5 wt% CNC-CNF reinforcements, indicating that the nanocelluloses were not well dispersed in the membrane and accumulated on the surface. Moreover, the number of agglomerates on the surface increased with the increase in the amount of nanocellulose incorporated into the membrane. In CNC and CNF reinforcements, the number of agglomerates on the membrane surface increased as the amount of nanocellulose incorporated into the membrane increased. In casting solutions where hydrophobic polymers are present, hydrophilic nanocellulose such as CNC and CNF may be poorly dispersed, which may lead to an increase in the total solid content of the casting solution [69]. The hydrophilic nanomaterials in the membrane casting solution tend to migrate towards the upper part during the immersion phase of the non-solvent-induced phase inversion method [41]. Therefore, it is possible to observe CNC and CNF agglomeration on the surface of the final membrane when a high amount of nanomaterials is present in the membrane casting solution. PSf/PVP/CNF-1 was the membrane with the most agglomerates and defects on the surface. Agglomerates and defects on the membrane surface can lead to clogging of the pores and disruption of water flow, leading to reduced filtration efficiency.

### 3.3. AFM Images and Roughness of Membranes

**Figure 5:**
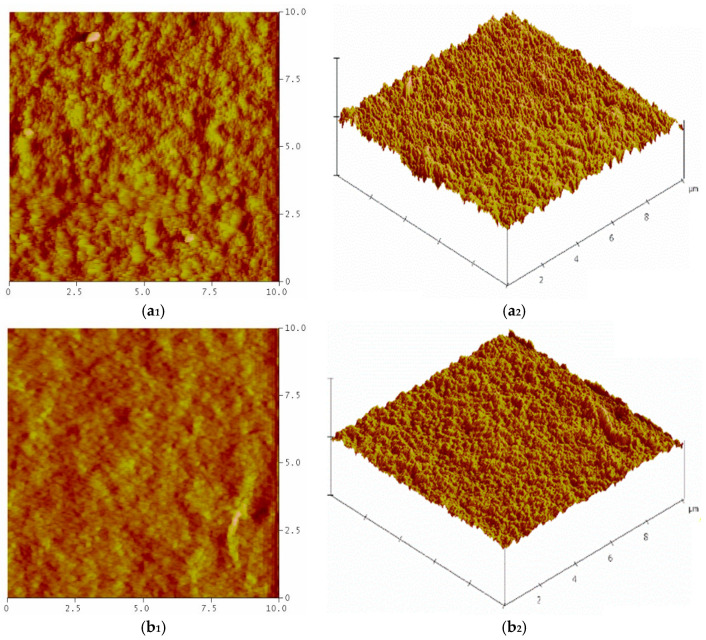

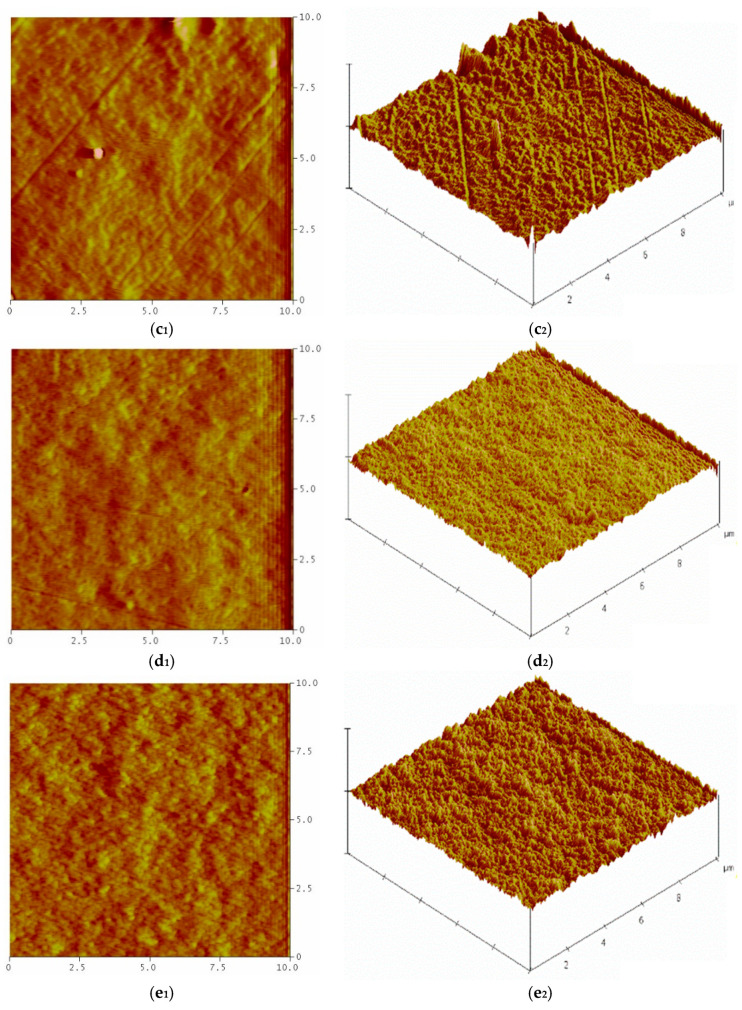

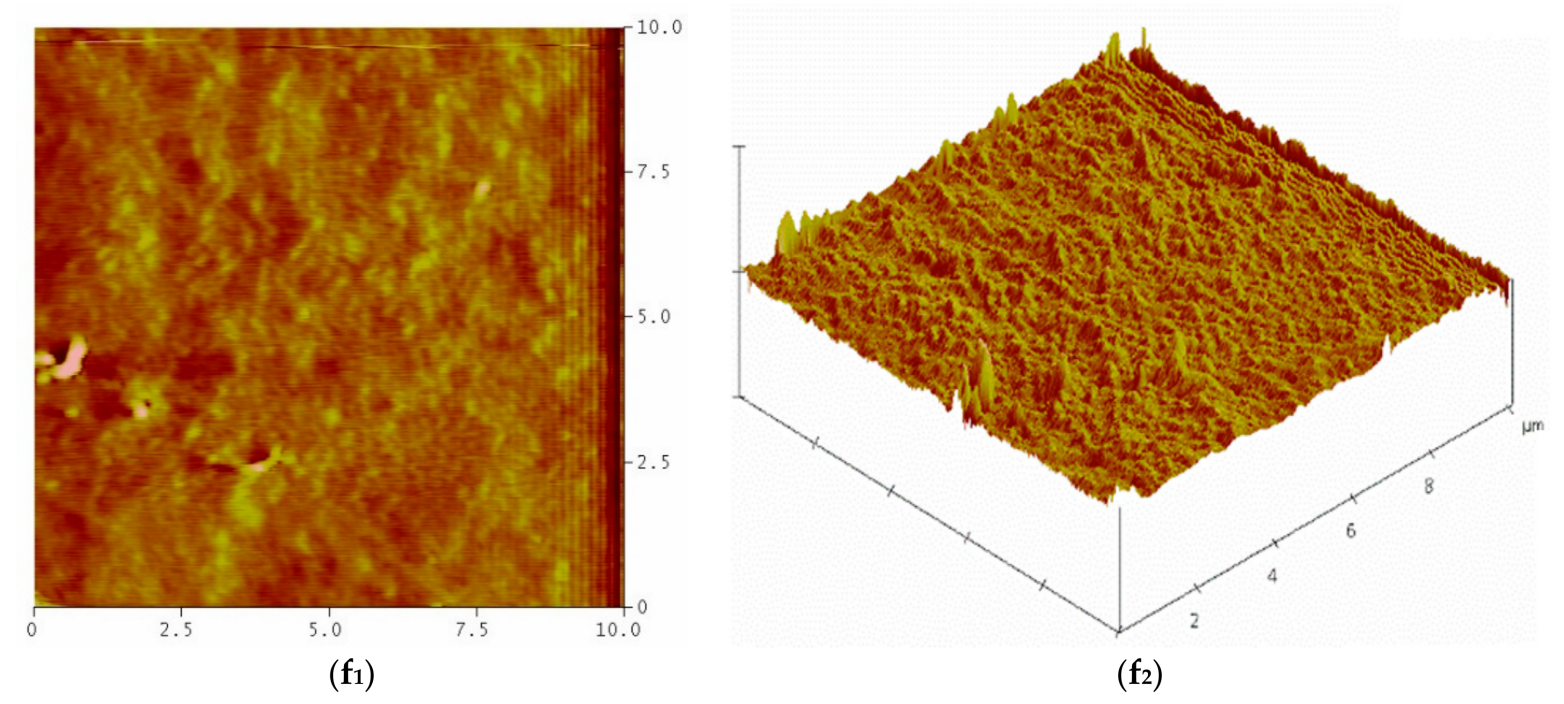
(1) Two-dimensional and (2) three-dimensional AFM images of PSf/PVP-based membranes: (**a_1_**,**a_2_**) PSf/PVP, (**b_1_**,**b_2_**) PSf/PVP/CNC-0.5, (**c_1_**,**c_2_**) PSf/PVP/CNC-1, (**d_1_**,**d_2_**) PSf/PVP/CNF-0.5, (**e_1_**,**e_2_**) PSf/PVP/CNF-1, and (**f_1_**,**f_2_**) PSf/PVP/CNC-CNF.

The surfaces of the produced membranes were further characterised by AFM analysis, and the roughness parameters of the membranes were determined. Figure 5 shows 2D and 3D AFM images of the membranes, and Table 2 shows the roughness parameters of the membranes. AFM images of all membranes showed that the membranes had a “peak-and-valley” structure. In the AFM images, the bright and dark regions are indicative of the peaks and valleys, respectively, on the surface of the membranes. The PSf/PVP membrane had the roughest surface, and the surface roughness of the membrane decreased with the addition of CNC, CNF, and CNC-CNF into the PSf/PVP membrane. Similarly, the roughness of the PES membrane decreased with the addition of CNC functionalised with amino acid cysteine to the membrane [70].

The PSf/PVP membrane had the highest Ra, Rrms, and Rz values, which were 5.94, 7.80, and 31.24 nm, respectively. The high surface porosity of the PSf/PVP membrane led to the formation of indentations and protrusions on the membrane surface, resulting in high membrane surface roughness. The Ra, Rrms, and Rz values of nanocomposite nanocellulose-reinforced membranes varied in the ranges of 1.40–2.59, 1.97–3.24, and 8.15–13.27 nm, respectively. As the proportion of CNC and CNF in the membrane increased from 0.5 wt% to 1 wt%, cracks and agglomerates increased on the membrane surface, resulting in an increase in the surface roughness of the membrane by 19.78% and 82.39%, respectively. The highest Ra, Rrms and Rz values belonged to PSf/PVP/CNF-1 membrane among the nanocomposite nanocellulose-reinforced membranes. This can be explained by the presence of more defects and agglomerates on the surface of the PSf/PVP/CNF-1 membrane than the other membranes. The PSf/PVP/CNC-CNF membrane exhibited a smoother surface due to the lower surface porosity and/or agglomerates on the surface compared to other membranes.

### 3.4. XRD Patterns of Membranes

The presence of amorphous materials in membranes, the presence of crystalline impurities, and the effect of various changes in membrane production on the membrane structure can be examined with XRD analysis [71].

**Figure 6:**
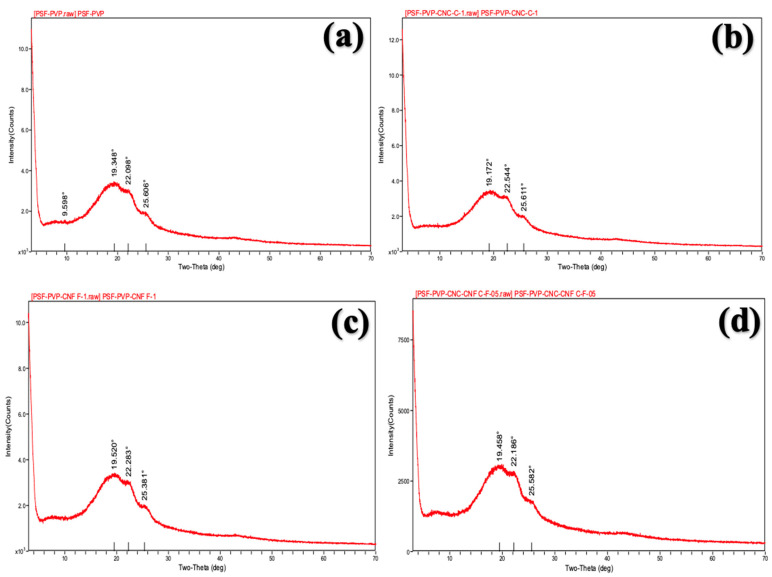
XRD patterns of PSf/PVP-based membranes: (**a**) PSf/PVP, (**b**) PSf/PVP/CNC-1, (**c**) PSf/PVP/CNF-1, and (**d**) PSf/PVP/CNC-CNF.

Figure 6 shows the XRD patterns of PSf/PVP and nanocellulose-reinforced nanocomposite PSf/PVP-based membranes. In the XRD patterns of PSf/PVP-based membranes, the broad peak at 2θ = 9–30° showed that the membranes were amorphous. In all PSf/PVP-based membranes, three peaks were detected in the broad peak at approximately 19°, 22°, and 25° at 2θ. Previous studies have revealed the amorphous structure of PSf membranes [71,72,73]. For instance, in a previous study, the broad peak in the range of 17–30° at 2θ in the XRD pattern of a pure PSf membrane revealed the amorphous structure of PSf [73]. In the study, in the PVP-added PSf membrane, unlike the pure PSf membrane, the peak at 2θ = 21.8° in the XRD pattern was attributed to the amorphous structure of PVP [73]. In this study, the amorphous structure of PSf/PVP-based membranes, which was elucidated by evaluating the XRD pattern of PSf/PVP-based membranes, was consistent with other studies.

Semi-crystalline materials are harder, and they have high chemical and thermal resistance [74]. On the other hand, amorphous materials are more flexible, and they have high impact resistance [74]. The incorporation of 1 wt% CNC, 1 wt% CNF, and 0.5 wt% CNC-CNF in the PSf/PVP membrane did not lead to any significant difference in the XRD pattern of the membrane and the intensity of the peaks. The absence of a significant change in the position and intensity of the peaks indicated that 1 wt% CNC, 1 wt% CNF, and 0.5 wt% CNC-CNF reinforcement did not change the crystalline structure of the membrane, and the existing crystalline structure was preserved. However, it is worth noting that PSf/PVP membranes exhibit good water flux performance, mechanical strength, separation efficiency, and fouling resistance without the significant need for the excellent resistance and stiffness provided by crystalline nanomaterials [75,76]. The addition of amorphous PVP [77] into a PSf membrane increases porosity, hydrophilicity, separation performance, and resistance to fouling of the PSf membrane [78]. Although CNC and CNF reinforcement did not contribute to increasing the crystallinity of PSf/PVP-based membranes, amorphous PSf/PVP membranes can be used in water treatment applications due to their superior performance.

### 3.5. Porosity and Average Pore Size of Membranes

**Figure 7:**
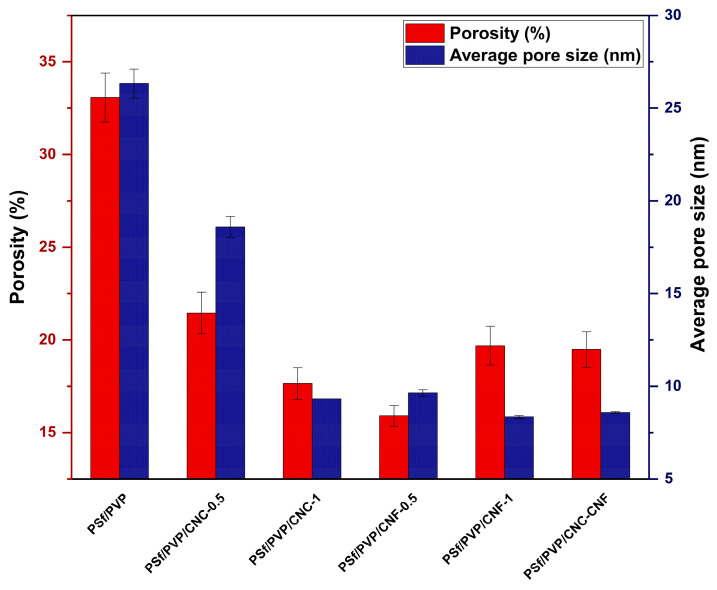
Porosity and average pore size of PSf/PVP-based membranes.

Porosity and pore size play an important role in the water flux and separation efficiency of membranes [79]. Figure 7 shows the porosity and average pore size of the membranes. PSf/PVP membrane had the highest porosity (33.08 ± 1.32%), and the porosity of the membrane decreased with nanocellulose reinforcement. The porosity of nanocellulose-reinforced nanocomposite membranes varied between 15.90 ± 0.56 and 21.45 ± 1.12%. Consistent with the SEM images, the membrane with the highest surface porosity was PSf/PVP. High porosity in membranes is a property that offers both advantages and disadvantages. The increased porosity of the membrane facilitates the passage of water and thus contributes to the high water flux observed during the filtration process. Furthermore, the filtration of water through high porosity membranes is facilitated, thus enabling high water flux at lower pressures [80]. Therefore, high porosity enables an energy-efficient filtration process. Conversely, high porosity can reduce the separation efficiency of the membrane as it increases the potential for contaminants in the water to more easily pass through the membrane. Furthermore, high porosity causes a reduction in the mechanical strength of the membrane, as the structural integrity of membranes with high porosity is less than that of membranes with lower porosity.

While the average pore size of PSf/PVP membrane was 26.33 ± 0.78 nm, the average pore sizes of PSf/PVP-based nanocomposite membranes varied between 8.34 ± 0.08 and 18.59 ± 0.56 nm. Since the average pore sizes of PSf/PVP-based membranes were 1–100 nm, it was determined that the membranes were UF membranes. While the highest average pore size was in the PSf/PVP membrane, the average pore size of the membrane decreased with CNC, CNF, and CNC-CNF reinforcement. A low amount (0.5 wt%) of CNF incorporation outperformed a low amount of CNC incorporation in reducing the porosity and average pore size of the membrane. Since the viscosity of the casting solution containing 0.5 wt% CNF was higher than that containing 0.5 wt% CNC, the liquid–liquid exchange during phase inversion was slower for the CNF-reinforced polymeric film, leading to a denser surface, lower porosity, and lower average pore size of the membrane.

### 3.6. Mechanical Properties of Membranes

**Figure 8:**
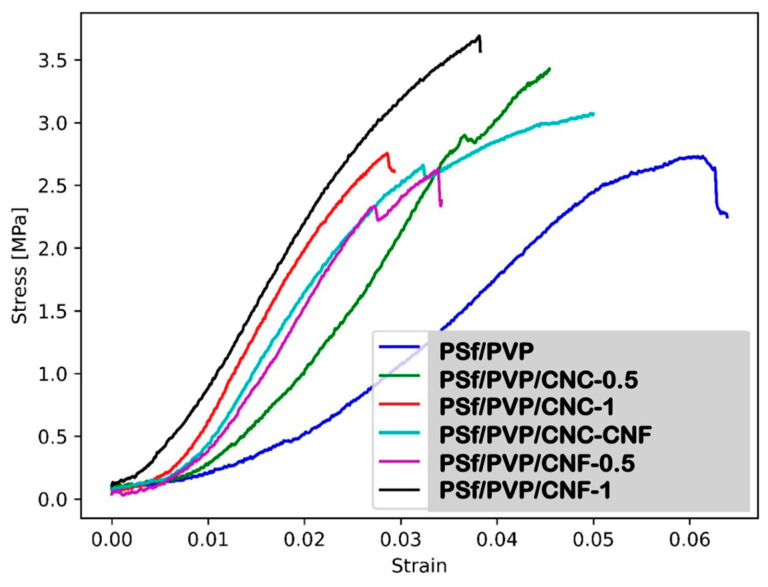
Average stress–strain curves of PSf/PVP-based membranes.

UF membranes operate under pressure in water and wastewater treatment, and these membranes must have mechanical strength that can overcome the applied pressure to serve the filtration process for a long time. Insufficient mechanical strength can lead to deformation and rupture of the membrane in the filtration process. Sharp particles in water and wastewater can damage membranes that do not have sufficient mechanical strength. In this study, the stress–strain curves, elasticity modulus, tensile strength, and elongation at break of membranes were investigated by tensile testing to investigate the mechanical properties of the membranes produced. Figure 8 shows the average stress–strain curves of the PSf/PVP-based membranes. All polymer-based membranes produced exhibited plastic deformation after elastic deformation. When the slopes of the membranes were compared in the stress–strain curve, the lowest slope was found for the PSf/PVP membrane, while the slopes of the membranes in the stress–strain graph increased with nanocellulose reinforcement of the membranes.

**Figure 9:**
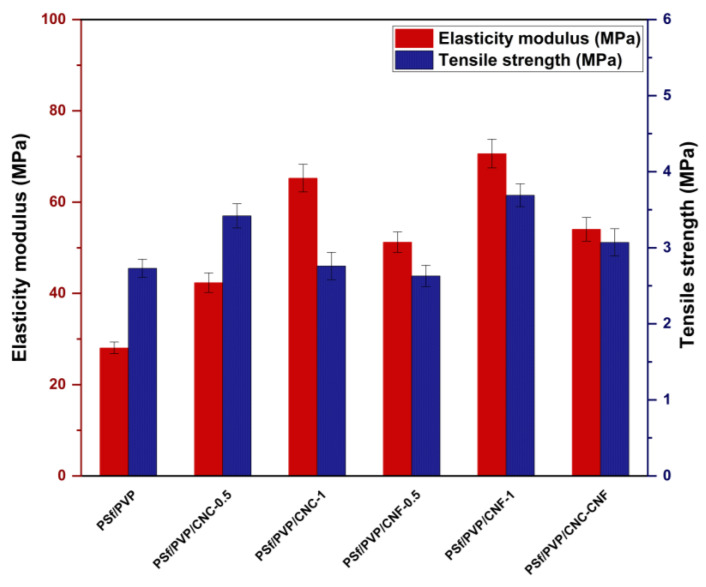
Elasticity modulus and tensile strength of PSf/PVP-based membranes.

**Figure 10:**
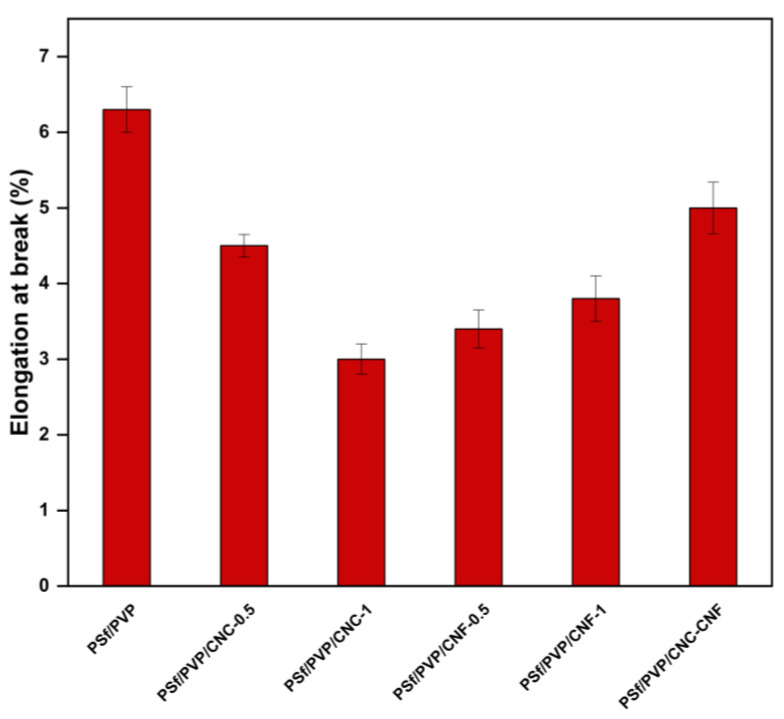
Elongation at break values of PSf/PVP-based membranes.

The elasticity modulus of the membranes was calculated by the ratio of stress to strain in the region where the membranes exhibit elastic behaviour in the stress–strain curve. Figure 9 shows the elasticity modulus and tensile strength of PSf/PVP-based membranes. The PSf/PVP membrane had the lowest elasticity modulus with 28.06 ± 1.3 MPa, while the elasticity modulus of the membrane increased with CNC, CNF, and CNC-CNF reinforcement. This can be attributed to the fact that the elasticity modulus of CNC and CNF is higher than the elasticity modulus of the membrane matrix materials [31,78]. As the amount of CNC and CNF incorporated into the PSf/PVP membrane was increased from 0.5 to 1 wt%, the membrane exhibited enhanced elasticity modulus. CNF performed better than CNC in increasing the rigidity of the polymer-based membrane. The addition of 0.5 wt% and 1 wt% CNC to the PSf/PVP membrane increased the elasticity modulus of the membrane by approximately 51% and 133%, respectively, while the addition of 0.5 wt% and 1 wt% CNF increased it by 83% and 152%, respectively. Among the membranes produced, the PSf/PVP/CNF-1 membrane had the highest elasticity modulus (70.63 ± 3.15 MPa) and the highest tensile strength (3.69 ± 0.15 MPa). This showed that the PSf/PVP/CNF-1 membrane was the most rigid and could withstand more stress. Figure 10 shows the elongation at break values of membranes. The elongation at break of the PSf/PVP membrane (6.3 ± 0.3%) decreased with CNC, CNF, and CNC-CNF reinforcement of the membrane. This indicated that nanocellulose-reinforced nanocomposite PSf/PVP-based membranes were less flexible and less deformable than PSf/PVP membranes. Similarly, in a study by Lalia et al. (2014), when 2 wt% nanocrystalline cellulose was added to polyvinylidene fluoride-co-hexafluoropropylene (PVDF-HFP) membrane, the tensile strength of the membrane increased from 12.6 to 17.2 MPa, while the modulus of elasticity of the membrane increased from 72 MPa to 105 MPa. On the other hand, in the study, 2 wt% nanocrystalline cellulose added to the PVDF-HFP membrane decreased the elongation at break of the membrane from 25 mm to 23 mm [81].

### 3.7. Comparison of Experimental Mechanical Properties of Membrane with Model Results

**Figure 11:**
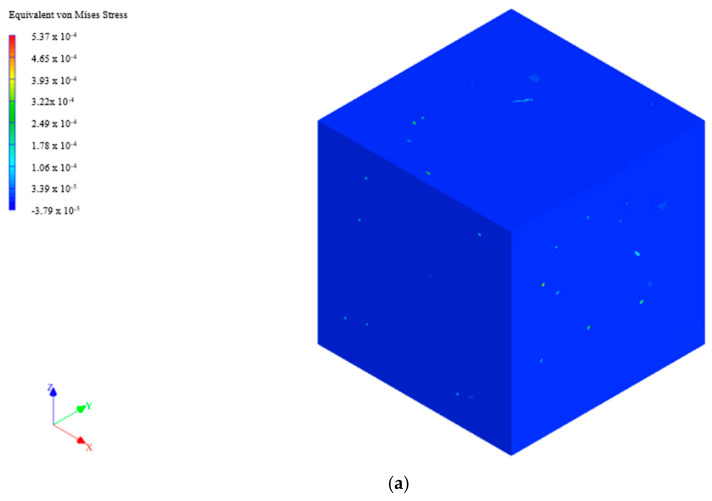

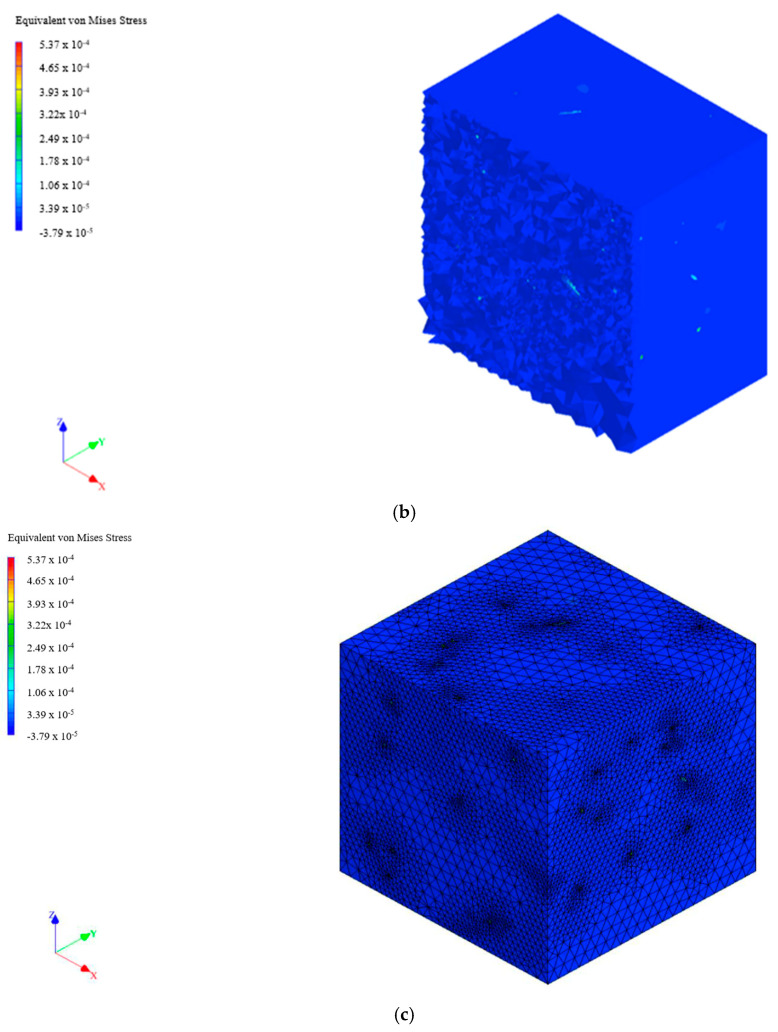

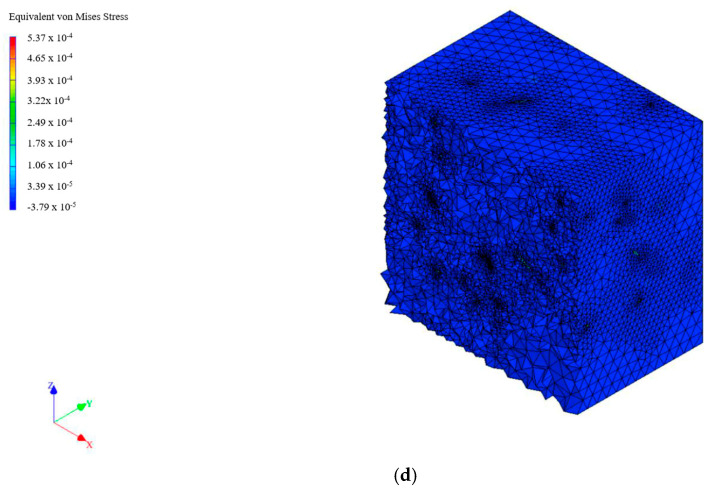
Equivalent von Mises stress distribution in RVE of PSf/PVP/CNC-CNF membrane: (**a**) whole RVE, (**b**) cross-section of the RVE, (**c**) whole RVE with mesh structure, and (**d**) cross-section of the RVE with mesh structure.

Table 3 shows the elasticity modulus of PSf/PVP-based nanocomposite membranes obtained from tensile tests and modelling approaches. The finite element was the most successful method in estimating the experimental modulus of elasticity values of nanocellulose-reinforced PSf/PVP-based membranes. Although the results obtained from the finite element method were quite close to the experimental data obtained from the tensile test of the nanocomposite membranes, the mechanical analysis of each membrane with the finite element approach took longer, approximately 8 h for each membrane. Nevertheless, despite the necessity for longer analysis times when utilising the finite element method to examine the mechanical properties of membranes, this approach offers the distinct advantage of enabling the visual examination of the equivalent von Mises stress distribution of the membrane. For instance, Figure 11 shows the equivalent von Mises stress distribution in a whole RVE, cross-section of the RVE, whole RVE with mesh structure, and cross-section of the RVE with mesh structure, which were generated for the analysis of the mechanical properties of the CNC-CNF-reinforced PSf/PVP membrane by the finite element method. As mentioned before, the density of the mesh structure was increased to perform more precise calculations in the vicinity of the nanomaterials in the membrane matrix in the modelling with the finite element approach. The von Mises equivalent stress values were higher on nanomaterials in both whole RVE and cross-sectional RVE. The nanomaterials corresponded to lighter colors such as green and yellow, representing higher equivalent von Mises stresses. This indicated that a significant portion of the load applied to the membrane was carried by the reinforcement materials. The modelling results demonstrated that the incorporation of nanomaterials (CNC and CNF) into the polymeric membrane matrix resulted in an enhancement of the membrane’s load-carrying capacity. As for other modelling methods, the computational Voigt–Reuss method, self-consistent scheme, and Halpin–Tsai methods were not successful in estimating the elasticity modulus of membranes with a very low error margin. The Mori–Tanaka mean-field homogenisation method was relatively successful in estimating the experimental elasticity modulus of membranes, although it did not provide visual results like the finite element method. One of the most important advantages of the Mori–Tanaka method was that the mechanical analysis of each membrane took approximately 10 min.

### 3.8. Water Flux Performance of Membranes

**Figure 12:**
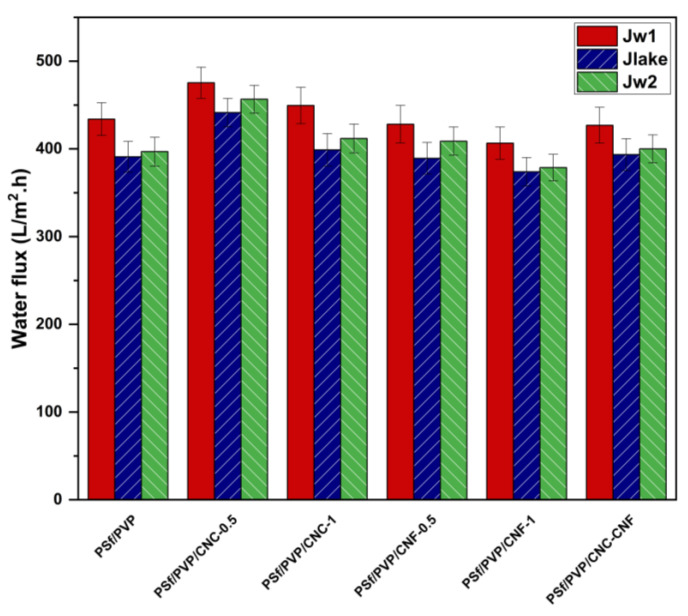
Water fluxes of PSf/PVP-based membranes at 3 bar.

The water flux performance of membranes is a critical parameter that affects the efficiency of the filtration process, energy efficiency, and the size of equipment required for filtration [82,83]. Membranes with higher water flux performance allow more water to be filtered in less time [53]. Membranes with high water flux performance require less energy consumption for water filtration, reducing the process’s operating cost [84]. A filtration process using membranes with high water flux performance requires less membrane surface area than a filtration process using membranes with lower flux performance, reducing both the cost of equipment (membranes and equipment) and the space occupied by the membrane system [85]. Figure 12 shows the water fluxes of PSf/PVP-based membranes. While the flux of PSf/PVP membrane for distilled water was 433.92 ± 18.69 L/m^2^.h at 3 bar, the water flux performance of the membrane increased by 9.58% and 3.60% with a 0.5% CNC and 1% CNC reinforcement in the membrane, respectively. Bai et al. (2017) also reported that while the pure water flux of a polyethersulfone (PES) membrane at 0.5 MPa was 1240 L/m^2^.h, the pure water flux of the membrane increased to 3524 L/m^2^.h with 2 wt% CNC reinforcement at the same pressure. In another study, 0.5 wt.% CNC reinforcement yielded a pure water flux increase from 2.5 to 10.6 L/m^2^.h and from 0 to 2.6 L/m^2^.h for membranes based on 20 wt.% PSf and 30 wt.% PSf produced using the immersion precipitation technique, respectively [86]. On the other hand, the flux of the membrane for distilled water decreased by 1.32% and 6.30%, respectively, with 0.5% and 1% CNF by weight reinforcement of the PSf/PVP membrane. The fact that the 0.5 wt% CNC-reinforced membrane exhibited the highest distilled water flux performance (475.50 ± 17.77 L/m^2^.h) among all membranes can be attributed to (1) the high surface porosity of the membrane (Figure 4b), (2) the agglomerate-free surface of the membrane (Figure 4b), and (3) the presence of hydrophilic CNCs on the membrane surface that facilitate the passage of water through the membrane. The flux performance of the membranes decreased due to the lower surface porosity and smaller pore sizes of highly CNC-, CNF-, and CNC-CNF-reinforced membranes. In these membranes, the agglomerates of nanocellulose blocked the pores on the surface, which may have prevented the water from filtering more uniformly through the membrane under pressure and may have caused the flux reduction.

The flux performance of all membranes for lake water was lower than that for distilled water since lake water contains organic and inorganic pollutants and the pollutants in lake water accumulate in the pores and surface of the membrane. The trend observed for distilled water flux performance of the membranes was also observed for lake water flux performance. While the flux of PSf/PVP membrane for lake water was 391.09 ± 17.54 L/m^2^.h at 3 bar, the flux of the membrane for lake water increased by 12.87% and 2.01% with 0.5% and 1% CNC by weight, respectively. However, the flux performance of the membrane for lake water decreased with CNF addition.

The distilled water fluxes of the membranes cleaned by physical cleaning were higher than the lake water fluxes of the membranes. However, the distilled water fluxes of the fouled–cleaned membranes were lower than the distilled water flux of the clean membranes. This indicated that the pollutants accumulated and/or weakly adsorbed on the membrane surface and/or pores during lake water filtration could not be completely removed by physical cleaning, but only a part of them could be removed. While the distilled water flux of the fouled–cleaned PSf/PVP membrane was 396.90 ± 16.45 L/m^2^.h, the flux of the membrane increased with low and high CNC, low CNF, and CNC-CNF reinforcement. This result indicated that CNC, low CNF, and CNC-CNF reinforcement contributed to weaker attachment of pollutants in water to the surface/pores of the membrane and more effective removal of pollutants from the membrane by the hydraulic cleaning method.

### 3.9. UV254 and TOC Removal Performance of Membranes

**Figure 13:**
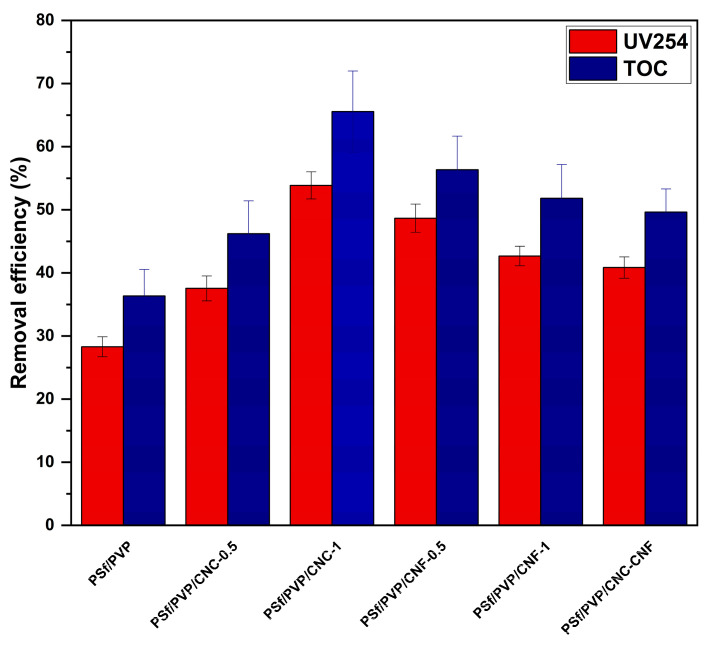
UV254 and TOC removal efficiency of PSf/PVP-based membranes.

During the disinfection process in drinking water treatment, organic matter in the water interacts with disinfectants such as chlorine and causes the formation of disinfection by-products that pose a threat to human health [87]; therefore, it is necessary to effectively remove organic matter from water. Figure 13 shows the UV254 and TOC removal efficiency of PSf/PVP-based membranes from lake water. The UV254 and TOC values of lake water were 0.148 cm^−1^ and 6.12 mg/L, respectively. The UV254 and TOC removal efficiency of PSf/PVP membrane from lake water was low with 28.30 ± 1.58% and 36.35 ± 4.20% efficiency, respectively. The UV254 and TOC removal efficiencies of the nanocomposite membranes varied in the ranges of 37.55 ± 1.98%–53.87 ± 2.15% and 46.22 ± 5.20%–65.56 ± 6.44%. The decrease in porosity and pore size of nanocellulose-reinforced membranes compared to PSf/PVP membranes contributed to the increase in organic matter removal efficiency from water. Among the nanocellulose-reinforced nanocomposite membranes, the PSf/PVP/CNC-1 membrane exhibited the highest UV254 and TOC removal efficiency with 53.87 ± 2.15% and 65.56 ± 6.44%, respectively. The results revealed that CNC, CNF, and CNC-CNF reinforcement contributed to improving the UV254 and TOC removal performance of the membrane by modifying the physical properties. Currently, there are no studies on the removal of TOC and UV254 from lake water using nanocellulose-reinforced polymer-based membranes. Some studies have reported that BSA [36,88], iron [89], chromium [89], and turbidity [89] removal increased by modifying polymer-based membranes with nanocellulose.

### 3.10. Antifouling Ability of Membranes

**Figure 14:**
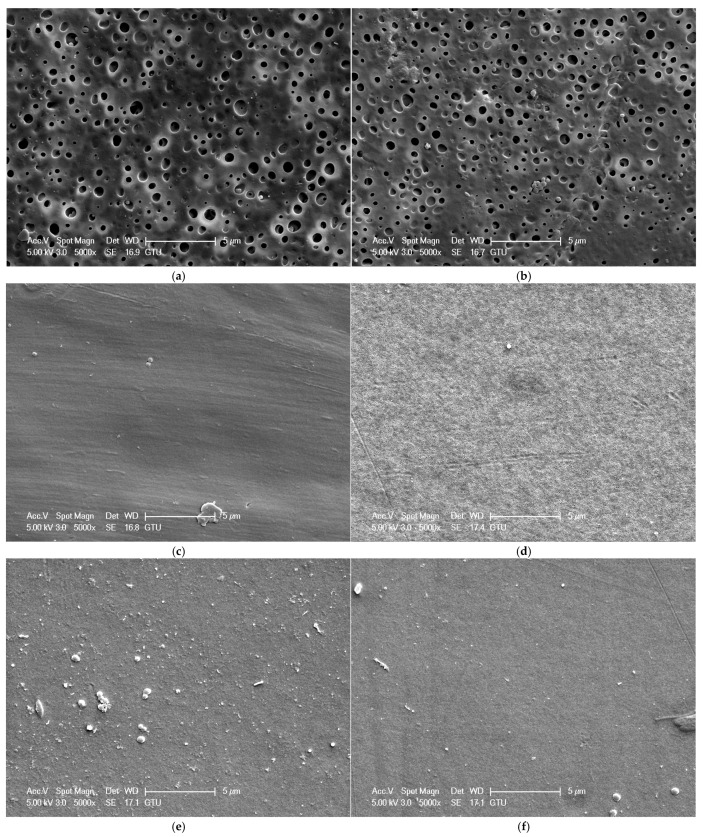
SEM surface images of fouled–cleaned PSf/PVP-based membranes: (**a**) PSf/PVP, (**b**) PSf/PVP/CNC-0.5, (**c**) PSf/PVP/CNC-1, (**d**) PSf/PVP/CNF-0.5, (**e**) PSf/PVP/CNF-1, and (**f**) PSf/PVP/CNC-CNF.

To further investigate the resistance of membrane surfaces to fouling, SEM surface images of membranes physically cleaned for 15 min after lake water filtration (fouled–cleaned membranes) were examined (Figure 14). The high surface porosity of the fouled–cleaned PSf/PVP and PSf/PVP-CNC-0.5 membranes and the dense surfaces of other fouled–cleaned membranes were consistent with the surface images of the clean membranes produced. Even after the membranes were used in lake water filtration and physically cleaned, pollutants were observed to accumulate on their surface. Pollutants on PSf/PVP/CNC-0.5 and PSf/PVP/CNF-0.5 membranes were less than those on PSf/PVP/CNC-1 and PSf/PVP/CNF-1 membranes. This indicated that the surface of the membranes with a lower proportion of nanocellulose reinforcement was more resistant to fouling. Among the nanocellulose-reinforced membranes produced, the PSf/PVP/CNC-0.5 membrane accumulated fewer pollutants on the surface than the other membranes, and most of the surface pores of the PSf/PVP/CNC-0.5 membrane were open.

**Figure 15:**
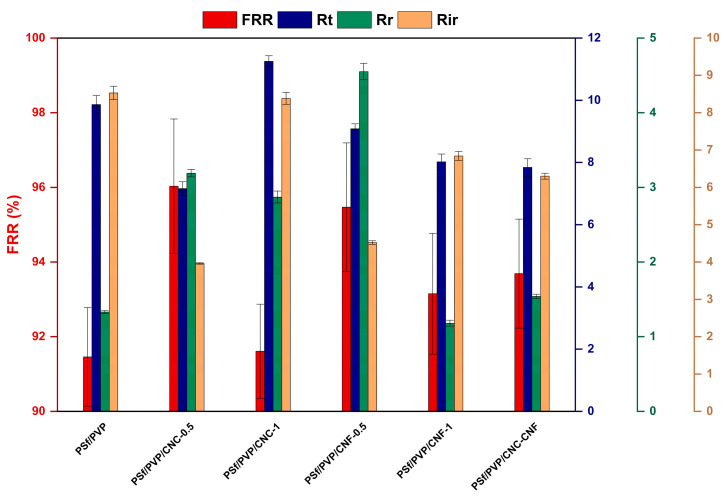
FRR values and fouling parameters of PSf/PVP-based membranes.

The FRR, Rt, Rr, and Rir values of the membranes were also calculated to better understand the membrane fouling behaviour. A higher FRR value indicates that the membrane has superior resistance to fouling. Figure 15 shows the FRR value and fouling parameters of PSf/PVP-based membranes. The FRR value of the PSf/PVP membrane was 91.46%, and the FRR value of the membrane increased with CNC and CNF reinforcement in the PSf/PVP membrane. The FRR of the membranes at lower CNC and CNF reinforcement ratios was higher than that at higher reinforcement ratios. In a study by Zhang et al. (2018), the antifouling properties of PES/PVP and PES/PVP/CNC membranes were investigated after filtration of bovine serum albumin/water solution. In the study, when the amount of CNC in the PES/PVP membrane increased from 0 to 5 wt%, the FRR value of the membrane increased from 51% to 90% [88]. Boruah et al. (2023) reported that while the FRR value of 15 wt% PVDF-based membrane was 40% in filtration using BSA solution, the FRR value increased to 70% with 0.3 wt% CNC reinforcement in the membrane. The Rt value of the PSf/PVP membrane was 9.86%, and Rir constituted most of the Rt value of the membrane. The Rt value decreased with 0.5 wt% CNC reinforcement of the PSf/PVP membrane, while the Rt value increased to 11.25% with 1 wt% CNC reinforcement. Fouling parameters and FRR values revealed that among the membranes produced, especially the PSf/PVP-based membrane with 0.5 wt% CNC reinforcement exhibited better resistance to fouling. Considering the SEM surface images, FRR values, and fouling parameters of the fouled–cleaned membranes, the membrane with the best antifouling ability among all membranes was PSf/PVP/CNC-0.5. A study showed that coating a PES membrane with CNC or CNF improved the membranes’ antifouling properties and alleviated reversible and irreversible fouling caused by humic acid and bovine serum albumin [90].

## 4. Conclusions

In this study, the effects of two different types of nanocellulose on the properties (morphology, roughness, amorphous structure, porosity, average pore size, mechanical properties), water flux performance, organic matter removal efficiency from water, and antifouling ability of PSf/PVP-based membranes produced by the phase inversion method were investigated. With CNC, CNF, and CNC-CNF reinforcement of PSf/PVP-based membranes, the porosity, average pore size, and surface roughness of the membranes decreased, while the organic matter removal efficiency from water (UV254 and TOC) and elasticity modulus of the membrane increased. CNC, CNF, and CNC-CNF reinforcements did not cause significant changes in the amorphous structure of the PSf/PVP membrane.

The PSf/PVP membrane fluxes for distilled water and lake water were 433.92 ± 18.69 L/m^2^.h and 391.09 ± 17.54 at 3 bar. CNC reinforcement was more effective in increasing the water flux performance of the membrane than CNF reinforcement. The flux performance of the membrane for distilled water and lake water increased by 9.58% and 12.87%, respectively, with 0.5 wt% CNC reinforcement in the PSf/PVP membrane. The PSf/PVP/CNC-0.5 membrane had the highest distilled water flux (475.50 ± 17.77 L/m^2^.h) among all the membranes produced, and its resistance to fouling was the highest.

Nanocellulose-reinforced nanocomposite membranes exhibited higher UV254 (37.55 ± 1.98%–53.87 ± 2.15%) and TOC (46.22 ± 5.20%–65.56 ± 6.44%) removal efficiencies than PSf/PVP membranes. Moreover, nanocellulose reinforcement improved the antifouling ability of PSf/PVP membranes. The level of foulants accumulated on the surface of PSf/PVP/CNC-0.5 and PSf/PVP/CNF-0.5 membranes was less than that on the surface of PSf/PVP/CNC-1 and PSf/PVP/CNF-1 membranes after lake water filtration and hydraulic membrane cleaning. PSf/PVP/CNC-0.5 was the most fouling-resistant membrane, with a high FRR and low Rt value.

Different numerical model approaches were evaluated to estimate the mechanical properties of nanocomposite membranes, and the finite element method was the most successful method to estimate the mechanical properties of the membrane. The Mori–Tanaka mean-field homogenisation method provided good results in estimating the mechanical properties of nanocellulose-reinforced nanocomposite membranes following the finite element method. Before starting the production of nanocomposite membranes with desired mechanical properties, the mechanical properties of the membranes can be predicted using software with the finite element and Mori–Tanaka mean-field homogenisation methods. This saves both time and material waste. Although the finite element method requires longer times to analyse PSf/PVP membranes, the finite element was the best method because it offers the advantage of high accuracy in estimating the mechanical properties of the membranes and visual inspection of the stress distribution in the membrane. On the other hand, the mechanical properties of PSf/PVP membranes can be estimated in much shorter times with a relatively low margin of error using the Mori–Tanaka mean-field homogenisation method. In the analysis of membranes, a choice between the two methods can be made by considering the factors of margin of error and speed of mechanical analysis.

## Figures and Tables

**Table 1:** Composition of casting solutions prepared for the production of PSf/PVP-based flat sheet membranes.

	PSf (% wt.)	PVP (% wt.)	DMF (% wt.)	CNC (% wt.)	CNF (% wt.)
PSf/PVP	20	5	75	-	-
PSf/PVP/CNC-0.5	20	5	74.5	0.5	-
PSf/PVP/CNC-1	20	5	74	1	-
PSf/PVP/CNF-0.5	20	5	74.5	-	0.5
PSf/PVP/CNF-1	20	5	74	-	1
PSf/PVP/CNC-CNF	20	5	74.5	0.25	0.25

**Table 2:** Surface roughness parameters of membranes.

	Ra (nm)	Rrms (nm)	Rz (nm)
PSf/PVP	5.94	7.80	31.24
PSf/PVP/CNC-0.5	1.82	2.38	10.44
PSf/PVP/CNC-1	2.18	2.97	13.08
PSf/PVP/CNF-0.5	1.42	1.99	8.15
PSf/PVP/CNF-1	2.59	3.34	13.27
PSf/PVP/CNC-CNF	1.40	1.97	8.39

**Table 3:** Elasticity modulus of PSf/PVP-based membranes: experimental and modelling results.

Membrane	Experimental Result	Mori–Tanaka	Finite Element	$E_{\mathrm{Voigt}}$	$E_{Reuss}$	Self-Consistent Scheme	Halpin–Tsai (Eeff,long)	Halpin–Tsai (Eeff,trans)
PSf/PVP	28.06	-	-	-	-	-	-	-
PSf/PVP/CNC-0.5	42.35	43.5	42.03	42.99	28.15	47.97	43.61	40.68
PSf/PVP/CNF-0.5	51.21	52.52	50.10	54.67	28.15	55.33	52.17	48.51
PSf/PVP/CNC-1	65.24	59.17	66.28	57.97	28.24	56.93	71.23	60.72
PSf/PVP/CNF-1	70.62	77.16	71.58	81.38	28.25	65.68	112.95	92.66
PSf/PVP/CNC-CNF	54.05	48.04	57.37	48.36	28.15	51.89	48.36	44.12

## Data Availability

All datasets related to this publication are accessible to the readers.
